# Effects of broadband music and audible band music on relaxation states and cognitive function in young adults: a randomized controlled trial

**DOI:** 10.1186/s40001-024-01943-z

**Published:** 2024-07-19

**Authors:** Lu Lin, Shufang Zuo, Yao Liu, Ito Masato, Machidori Wataru, Kumamoto Yasuhiro, Kakuhari Isao, Si Chen, Ziyu Wang, Cui Ye, Xuan Huang

**Affiliations:** 1 The First Affiliated Hospital of Soochow University, No. 188 Shizi Street, Suzhou, 215006 China; 2 School of Nursing, Suzhou Medical College of Soochow University, Suzhou, 215006 China; 3 System Solutions Development Center, Advanced Value Analysis Department, Panasonic R&D Center Suzhou Co., Ltd, Suzhou, 215123 China; 4 Planning and Administration Department, Product Analysis Center , Panasonic Holdings Corporation, Osaka, 571-8501 Japan; 5 Digital & AI Technology Center, Technology Division, Panasonic Holdings Corporation, Osaka, 571-8501 Japan

**Keywords:** Broadband, Audible band, Music, Relaxation, Cognitive function, Young adults

## Abstract

**Background:**

Although broadband music with inaudible high-frequency components may benefit human well-being, this research area is largely unexplored and lacks sufficient studies on the topic. This study aimed to investigate and compare the effects of broadband and audible band music on relaxation states and cognitive function in young adults.

**Methods:**

A single-blind randomized controlled trial was conducted in a professional soundproof laboratory from December 22, 2022, to January 18, 2023 with 32 participants randomly assigned to two groups, “Day 1 broadband + Day 2 audible band” (n = 16) and “Day 1 audible band + Day 2 broadband” (n = 16), listening to either broadband or audible band music (the same music piece played on the piano and harp) for two sessions of 15 min each on two consecutive days. Cognitive function was measured using CNS Vital Signs at pre-listening, after the 1st session, and after the 2nd session, while heart rate was monitored throughout the experiment. Visual Analog Scale was also administered for self-reported arousal, stress, thinking ability, and attention following each listening session.

**Results:**

No significant differences were found in heart rate, cognitive flexibility, and executive function between the broadband listening group and the audible band-listening group (*p* > 0.05). However, the broadband group exhibited significant differences in mean heart rate at several time points, as well as a significant improvement in VAS stress level during the 2nd listening session compared to the 1st (*p* < 0.05). On the other hand, significant improvements in cognitive flexibility and executive function were observed in the audible band group across different time points (*p* < 0.05).

**Conclusion:**

Comparative analysis showed that broadband and audible band music influenced cognitive function differently. Short-term audible band music listening significantly improved cognitive flexibility and executive function, while short-term broadband music listening significantly reduced reaction time in cognitive tests. Additionally, broadband music consistently resulted in lower mean heart rates compared to audible band music at all time points, suggesting that it may be more effective in promoting relaxation and reducing stress, although these differences were not statistically significant. Since the cognitive enhancing effects of broadband music may be counteracted by the drowsy effect of the selected relaxing music, using different types of music may be necessary to confirm its effects in future studies.

## Introduction

The human ear can perceive sound frequencies ranging from approximately 20 Hz to 20,000 Hz [1]. With advances in information and communication technologies, broadband audio, encompassing sounds both audible and inaudible to human ears, has emerged in the digital music market. Unlike conventional audible band audio, broadband audio has a higher sampling frequency and greater bit depth, resulting in a closer replication of real analog sound waves. A higher sampling frequency improves sound digitization accuracy in the time–frequency domain, while a greater bit depth increases sound resolution [2]. However, the benefits of broadband audio for humans have not been sufficiently investigated.

The conventional digital recording process eliminates inaudible high-frequency components, while broadband music that retains such components has been found to affect human electroencephalographic (EEG) activity, referred to as the “hypersonic” effect [3–5]. The influence of inaudible high-frequency components on the psychological and cognitive states of individuals is not well understood. Although full-range audio has been rated better in sound quality than high-cut audio, participants often cannot distinguish between the two types of digital audio [6]. Prior research has indicated that inaudible high-frequency components increase EEG alpha-band frequency power, which is related to arousal, vigilance levels, and cognitive tasks involving perception, working memory, long-term memory, and attention [7–11]. The presence of inaudible high-frequency components might affect sound perception and behavioral aspects by activating the brainstem and thalamus areas, which are involved in emotional experience and filtering or gating sensory input. Additionally, beta-band power has been shown to increase with corresponding increases in arousal and vigilance levels, indicating participant engagement in a task [12].

Some studies have demonstrated that adding inaudible high-frequency components to music has a positive impact on relaxation and cognitive function. By stimulating the brain without conscious awareness, the inaudible high-frequency components can improve mood, reduce stress, and enhance cognitive performance. This effect is achieved through a process known as “brainwave entrainment”, where the listener’s brainwaves synchronize with the frequency of the inaudible high-frequency components [2, 13].

While there is a possibility that broadband music containing high-frequency components that are not audible to humans could provide benefits for human well-being, this area of research remains largely unexplored and there is a dearth of studies on the topic. This study aimed to compare the effects of listening to broadband music and audible band music on the cognitive function and psychological states of young adults aged 20–30, using physiological, neuropsychological, and subjective measures. Specifically, we aimed to explore the impact of audible and broad bandwidths on the relaxation and cognitive function of study participants to gain insights into the potential applications of broadband music with inaudible high-frequency components for enhancing human well-being. The hypothesis for this study is: Compared with short-term listening to audible band music, short-term listening to broadband music results in a more significant improvement in relaxation levels and cognitive functioning in individuals aged 20–30.

## Methods

### Sound source

**Fig. 1:**
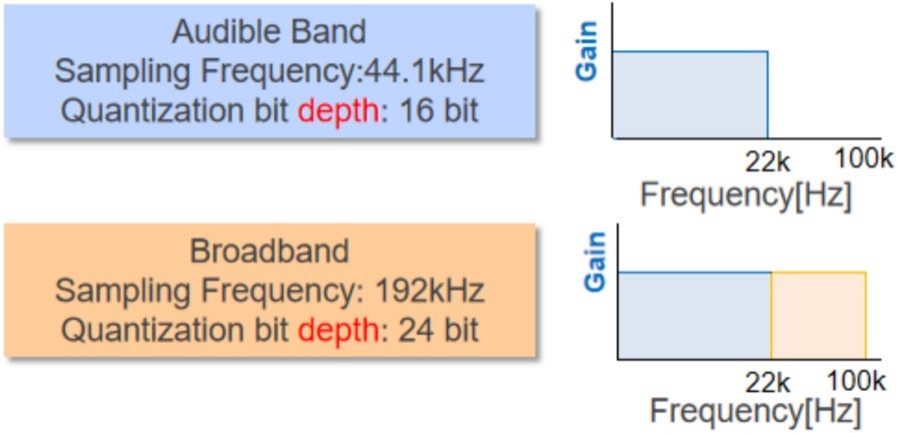
Frequency of the two sound sources used in the study

The broadband audio used in this study was a sound source with a high sampling frequency of 192 kHz and a high quantization bit depth of 24 bit. The audible band audio was a downsampled sound source with a lower sampling frequency of 44.1 kHz and a quantization bit depth of 16 bit. See Fig. 1. The playback hardware was a Technics SC-C500. The music piece used in this study was an existing sound file composed by Miwa Fukino, featuring harp and piano. It was pretested through a selection process where multiple license-free and high-resolution music pieces were evaluated in the laboratory with several participants. The participants’ subjective experiences of the music pieces were crucial in determining the properties of the music. All participants reported that this particular piece had a soothing effect during the listening experience. The music piece was 5 min long and was played on repeat three times to meet the 15-min experimental duration.

### Participants

For this study, 32 young adults aged between 20–30 years old were recruited as study participants according to the inclusion and exclusion criteria.

Inclusion criteria: (1) young adults aged 20–30 years; (2) voluntarily participate in this study and sign an informed consent form; (3) non-smoker.

Exclusion criteria: (1) currently receiving or have received other interventions that may affect the results of this study; (2) hearing or vision impairment that prevents cooperation with music listening or cognitive testing; (3) psychological disorders (such as depression) and use of antipsychotic medication; (4) severe cognitive impairment or diagnosed with a brain disease that affects cognitive function; (5) underlying diseases (such as hypertension, hyperlipidemia, hyperglycemia, arrhythmia, etc.); (6) currently pregnant or breastfeeding.

Additionally, to maintain consistency and control for potential confounding factors that may affect the outcome of the study, the participants were required to have meals 1.5 h prior to the start of the experiment. Consumption of alcoholic or caffeinated beverages was prohibited from 12 h prior to the start of the experiment.

### Design and procedure

**Fig. 2:**
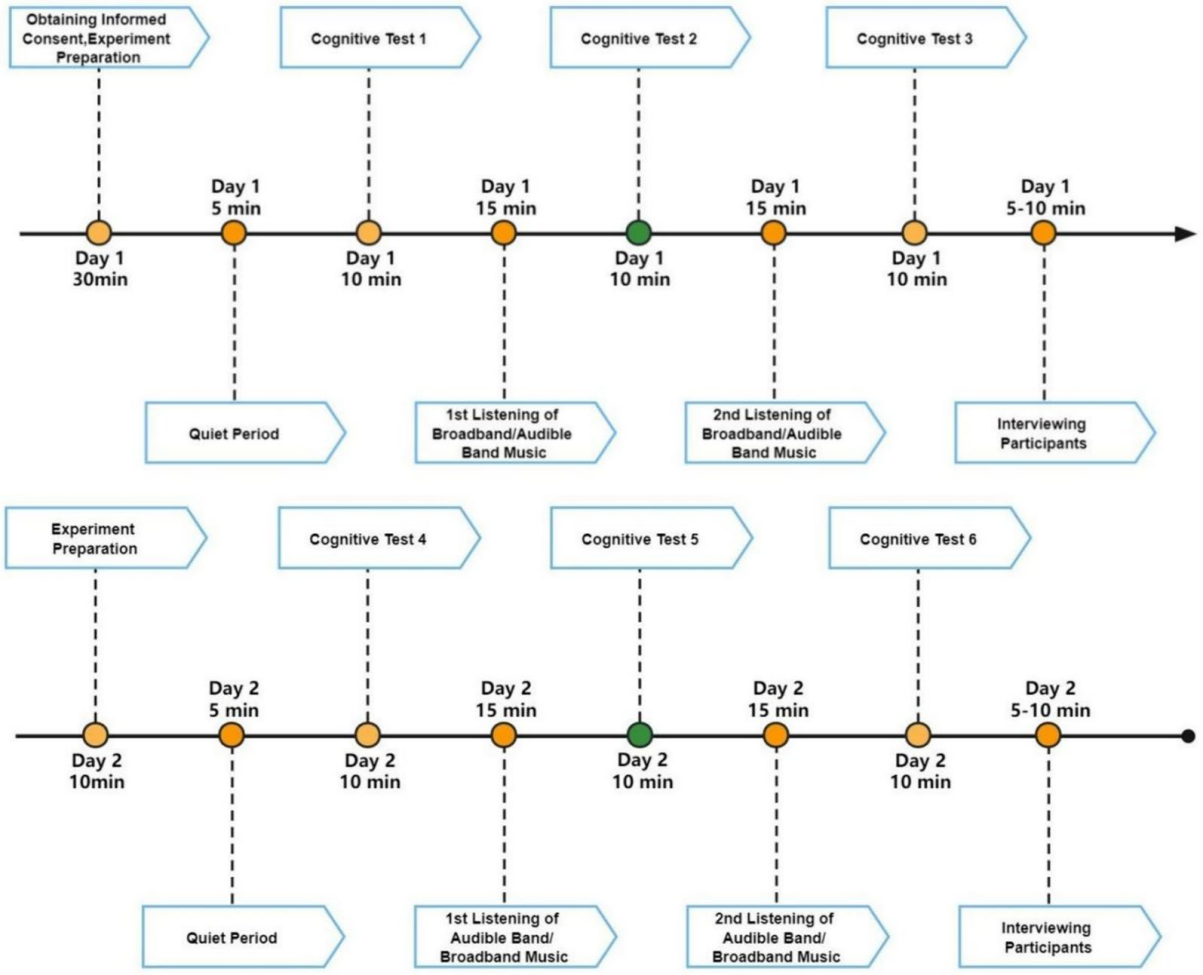
Flowchart of the experiment

A single-blind design was used, where the participants were unaware of the type of music being listened to. Using a random number table, the participants were randomly divided into two groups, “Day 1 broadband + Day 2 audible band” (hereinafter referred to as “D1B + D2A”) (n = 16) and “Day 1 audible band + Day 2 broadband” (hereinafter referred to as “D1A + D2B”) (n = 16). On Day 1, the “D1B + D2A” group listened to broadband music twice, while the “D1A + D2B” group listened to audible band music twice. On Day 2, the two groups switched the type of music they listened to. The participants were requested to keep their eyes open throughout the entire music listening session. The same music was listened to by both groups, and each participant listened to the music at the same time of day on both days. This indicates that the 32 participants listened to two separate sessions of broadband music and two separate sessions of audible band music. All the sessions took place during the day, starting at 9 am and ending at 5.30 pm each day. The flowchart of the experiment is shown in Fig. 2.

### Outcome measures

#### Heart rate

For heart rate measurement, the portable electrocardiography (ECG) of a versatile multi-sensor platform designed for raw biosignal acquisitions was used. Continuous measurements were taken from the end of the experiment preparation to the end of the final interview. The biosignals were converted into mean heart rates at a given time point using a custom program.

#### Cognitive function

In this study, cognitive function was measured by CNS Vital Signs (CNSVS), a global leader in designing and developing neurocognitive and behavioral assessment testing software for clinicians and researchers [14, 15]. The indicators of cognitive function included reaction time, cognitive flexibility, and executive function, which were assessed by two cognitive tests included in CNSVS, the Stroop Test and the Shifting Attention Test. After each listening session, the participants were required to complete cognitive function tests.

##### Reaction time

Reaction time refers to the time taken for the appearance of rapid voluntary reaction by an individual following a stimulus, either auditory or visual. In this study, it was measured by the Stroop Test in CNSVS [14, 15].

##### Cognitive flexibility

Cognitive flexibility refers to the ability to switch between different tasks, cognitive sets or mental processes in response to changing environmental demands. In this study, it was measured by both the Stroop Test and the Shifting Attention Test in CNSVS [14, 15].

##### Executive function

Executive function refers to complex cognitive processing requiring the coordination of several subprocesses to achieve a particular goal. In CNSVS, executive function tests assess the individual’s ability to process information, inhibit inappropriate responses, shift between tasks, and engage in abstract reasoning. In this study, the executive function of participants was measured by the Shifting Attention Test [14, 15].

#### Visual analog scale (VAS)

The study participants were asked to complete a Visual Analog Scale (VAS) to self-report their levels of arousal, stress, thinking ability, and attention following each music listening session. The VAS endpoint values ranged from 0 to 100, where higher scores indicated greater levels of arousal, thinking ability, and attention, and lower levels of stress.

### Statistical analysis

Statistical analysis was conducted using SPSS version 25.0 software (IBM Corporation, Armonk, NY, USA). Descriptive statistics were used to summarize demographic characteristics of the study population. Normality of data distribution was checked using the Shapiro–Wilk test. Continuous variables, being normally distributed, were reported as mean ± standard deviation (SD). Categorical variables were presented as frequencies and percentages. Since the same study population was divided into two subgroups based on the type and order of music listened to, a paired *t*-test was used for comparisons. A two-tailed *p* < 0.05 was considered statistically significant.

## Results

### Participants

The study participants were recruited between December 1 and December 15, 2022, and the experiment was conducted in a professional soundproof laboratory from December 22, 2022, to January 18, 2023. The characteristics of the 32 participants are detailed in Table 1.

**Table 1:** Demographics of participants (n = 32)

Item	n (%)
Age (years) (mean ± SD)	22.63 ± 2.31
Gender	
Male	12 (37.50%)
Female	20 (62.50%)
Level of education	
Undergraduate	14 (43.75%)
Postgraduate	18 (56.25%)
Major	
Science	20 (62.50%)
Arts	12 (37.50%)

### Heart rate

**Fig. 3:**
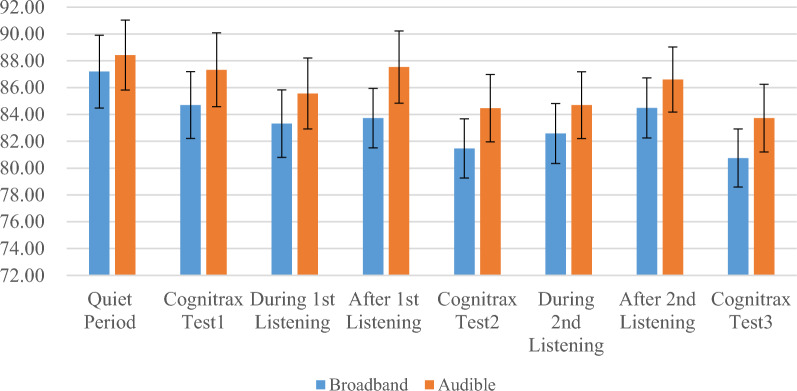
Mean heart rates in the broadband and audible band groups (n = 32) Cognitrax is a computerized testing system designed to measure and monitor brain performance, which is powered by CNS vital signs neurocognitive assessment technologies

When the participants were divided into two subgroups based on the type of music they listening to (broadband music or audible band music), no significant differences were observed in the mean heart rate between the two groups at eight different time points. Additionally, at all eight time points, the mean heart rate of the broadband group was lower than that of the audible band group. See Fig. 3. These eight time points included during the quiet time, Cognitive Test 1, during the 1st listening session, after the 1st listening session, Cognitive Test 2, during the 2nd listening session, after the 2nd listening session, and Cognitive Test 3. However, when looking at the data longitudinally, there were significant statistical differences in mean heart rate within the broadband group between quiet time and the 1st listening session (*p* < 0.01), quiet time and the 2nd listening session, quiet time and after the 1st listening session (*p* < 0.01), quiet time and after the 2nd listening session (*p* < 0.05), Test 1 and Test 2 (*p* < 0.01), and Test 1 and Test 3 (*p* < 0.01), with the former being significantly lower than the latter. Similarly, within the audible band group, there were significant statistical differences in mean heart rate between quiet time and after the 1st listening session (*p* < 0.01), quiet time and after the 2nd listening session (*p* < 0.01), during the 2nd listening session and after the 2nd listening session (*p* < 0.05), Test 1 and Test 2 (*p* < 0.01), and Test 1 and Test 3 (*p* < 0.01). See Table 2 for details.

**Table 2:** Mean heart rates in the broadband and audible band groups

Time point (mean ± SD)		Broadband (n = 32)	Audible band (n = 32)	Inter-group comparison	
				t	p
Quiet period		87.20 ± 12.73	88.43 ± 12.23	− 0.551	0.587
After 1st listening		83.72 ± 10.40	87.54 ± 12.63	− 1.601	0.124
Intra-group comparison	t	3.048	0.602		
	p	0.006**	0.553		
After 1st listening		83.72 ± 10.40	87.54 ± 12.63	− 1.601	0.124
After 2nd listening		84.49 ± 10.52	86.61 ± 11.38	− 1.113	0.278
Intra-group comparison	t	− 0.967	1.096		
	p	0.344	0.285		
Quiet period		87.20 ± 12.73	88.43 ± 12.23	− 0.551	0.587
After 2nd listening		84.49 ± 10.52	86.61 ± 11.38	− 1.113	0.278
Intra-group comparison	t	2.341	1.493		
	p	0.029*	0.15		
During 1st listening		83.32 ± 11.80	85.57 ± 12.41	− 1.008	0.325
After 1st listening		83.72 ± 10.40	87.54 ± 12.63	− 1.601	0.124
Intra-group comparison	t	− 0.443	− 1.406		
	p	0.663	0.174		
During 2nd listening		82.58 ± 10.49	84.69 ± 11.69	− 1.284	0.213
After 2nd listening		84.49 ± 10.52	86.61 ± 11.38	− 1.113	0.278
Intra-group comparison	t	− 1.733	− 2.179		
	p	0.098	0.041*		
Cognitrax test 1		84.70 ± 11.15	87.33 ± 12.91	− 1.092	0.287
Cognitrax test 2		81.47 ± 10.37	84.47 ± 11.76	− 1.864	0.076
Intra-group comparison	t	3.309	3.548		
	p	0.003**	0.002**		
Cognitrax test 2		81.47 ± 10.37	84.47 ± 11.76	− 1.864	0.076
Cognitrax test 3		80.75 ± 10.15	83.73 ± 11.85	− 1.636	0.117
Intra-group comparison	t	1.057	0.952		
	p	0.303	0.352		
Cognitrax test 1		84.70 ± 11.15	87.33 ± 12.91	− 1.092	0.287
Cognitrax test 3		80.75 ± 10.15	83.73 ± 11.85	− 1.636	0.117
Intra-group comparison	t	3.421	3.763		
	p	0.003**	0.001**		
Quiet period		87.20 ± 12.73	88.43 ± 12.23	− 0.551	0.587
During 1st listening		83.32 ± 11.80	85.57 ± 12.41	− 1.008	0.325
Intra-group comparison	t	3.154	3.091		
	p	0.005**	0.006**		
During 1st listening		83.32 ± 11.80	85.57 ± 12.41	− 1.008	0.325
During 2nd listening		82.58 ± 10.49	84.69 ± 11.69	− 1.284	0.213
Intra-group comparison	t	0.764	1.2		
	p	0.453	0.243		
Quiet period		87.20 ± 12.73	88.43 ± 12.23	− 0.551	0.587
During 2nd listening		82.58 ± 10.49	84.69 ± 11.69	− 1.284	0.213
Intra-group comparison	t	3.823	3.639		
	p	0.001**	0.002**		

Cognitrax is a computerized testing system designed to measure and monitor brain performance, which is powered by CNS Vital Signs neurocognitive assessment technologies

^*^p < 0.05; **p < 0.01

### Cognitive function

According to the results presented in Table 3, there were no statistically significant differences in cognitive flexibility, executive function, and reaction time between the two groups of participants who listened to broadband and audible band music before listening, after the 1st listening session, and after the 2nd listening session (all *p* > 0.05).

**Table 3:** Comparison of cognitive function between the broadband and audible band groups

Indicator (mean ± SD)	Time points	Broadband (n = 32)	Audible band (n = 32)	t p	
Cognitive flexibility	Pre-listening	51.08 ± 9.46	49.00 ± 7.76	0.862	0.397
	After 1st listening	52.36 ± 6.20	53.44 ± 6.24	− 0.696	0.493
	After 2nd listening	54.52 ± 6.26	56.12 ± 5.67	− 1.447	0.161
Executive function	Pre-listening	52.27 ± 8.90	49.80 ± 8.23	1.151	0.259
	After 1st listening	53.43 ± 6.31	53.93 ± 6.72	− 0.369	0.714
	After 2nd listening	55.27 ± 7.78	56.20 ± 6.35	− 0.765	0.450
Reaction time (ms)	Pre-listening	725.92 ± 105.80	723.27 ± 102.11	0.142	0.889
	After 1st listening	718.46 ± 107.83	726.35 ± 93.15	− 0.496	0.624
	After 2nd listening	719.69 ± 88.35	723.73 ± 100.08	− 0.351	0.728

**Fig. 4:**
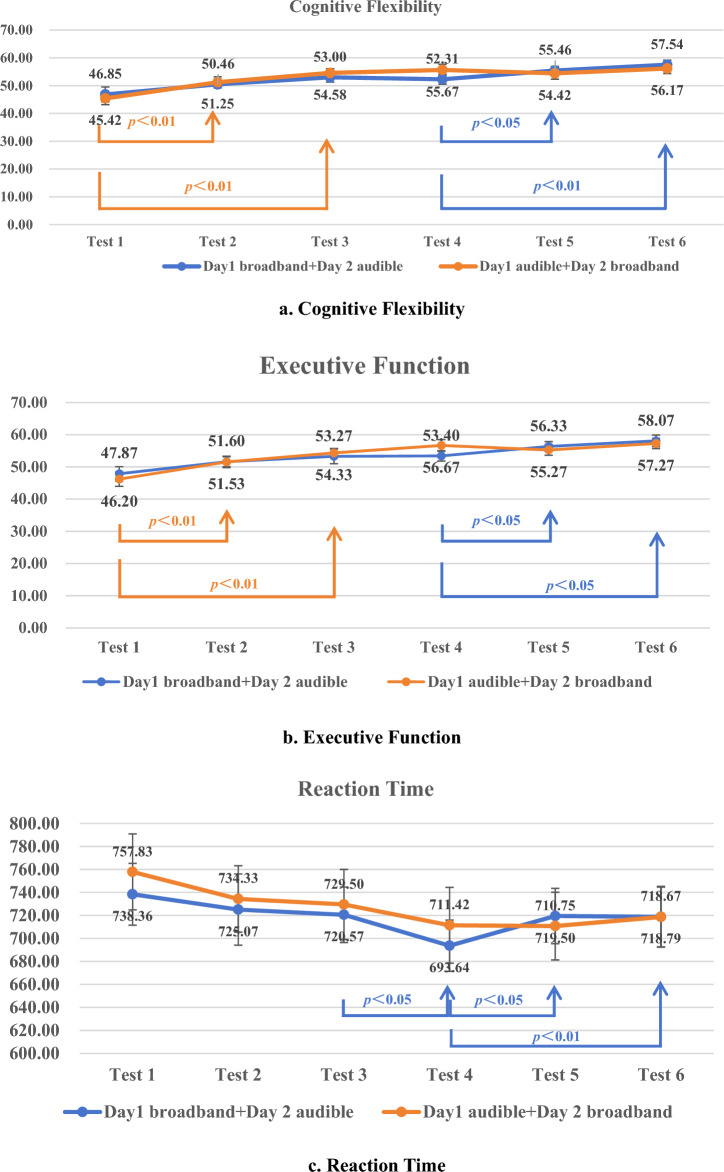
Day 1 Broadband + Day 2 Audible band vs. Day 1 Audible band + Day 2 Broadband in cognitive function (n = 16 + 16)

However, when the study population was divided into two groups, “D1B + D2A” and “D1A + D2B”, significant improvements were observed in cognitive flexibility and executive function scores between Test 4 (pre-listening) and Test 5 (after the 1st audible band listening session), as well as between Test 4 and Test 6 (after the 2nd audible band listening session) in the “D1B + D2A” group (*p* < 0.05). In addition, the “D1A + D2B” group showed significant improvements between Test 1 (pre-listening) and Test 2 (after the 1st audible band listening session), as well as between Test 1 and Test 3 (after the 2nd audible band listening session) in cognitive flexibility and executive function scores (*p* < 0.01). These results are shown in detail in Fig. 4a and Fig. 4b. Additionally, in this study, the minimum score for the cognitive flexibility test was 26 and the maximum score was 68, while the minimum score for the executive function test was 29 and the maximum score was 67.

Regarding reaction time, the “D1B + D2A” group showed a significantly lower reaction time in Test 4 (pre-listening) on Day 2 compared to Test 3 (after the 2nd broadband listening session) on Day 1 (*p* < 0.05), as well as a significantly longer reaction time in Test 5 (after the 1st audible band listening session) (*p* < 0.05) and Test 6 (after the 2nd audible band listening session) on Day 2 (*p* < 0.01) compared to Test 3 on Day 1. Further details can be found in Fig. 4c.

### VAS scores

The two groups of participants who listened to broadband and audible band music did not show significant statistical differences in their ratings for the four items of the VAS, including arousal, stress, thinking ability, and attention. However, when examining the longitudinal effects of music listening within each group, the broadband group showed a significant improvement in stress levels during the second listening session (84.50 ± 15.50) compared to the first listening session (79.93 ± 17.83) (*p* < 0.05). Meanwhile, there were no significant differences in arousal, thinking ability, and attention levels between the two listening sessions for the broadband group (*p* > 0.05). In contrast, the audible band group did not exhibit significant differences in VAS ratings for these four dimensions between the two music listening sessions (*p* > 0.05). See Table 4 for details.

**Table 4:** Music-induced psychological states using VAS in the broadband and audible band groups

Indicator (mean ± SD)			Broadband (n = 32)	Audible band (n = 32)	Inter-group comparison	
					t	p
Arousal	After 1st listening		60.13 ± 25.19	57.02 ± 22.53	0.767	0.449
	After 2nd listening		54.62 ± 25.57	50.38 ± 25.38	0.937	0.357
	Intra-group comparison	t	1.534	2.015		
		p	0.136	0.053		
Stress^a^	After 1st listening		79.93 ± 17.83	81.50 ± 15.28	− 0.715	0.48
	After 2nd listening		84.50 ± 15.50	85.53 ± 14.11	− 0.684	0.499
	Intra-group comparison	t	− 2.594	− 2.045		
		p	0.015^*^	0.05		
Thinking ability	After 1st listening		62.27 ± 23.41	62.68 ± 18.89	− 0.168	0.867
	After 2nd listening		59.72 ± 21.84	57.08 ± 25.30	0.768	0.449
	Intra-group comparison	t	0.769	2.045		
		p	0.448	0.05		
Attention	After 1st listening		66.45 ± 17.49	63.14 ± 19.46	1.235	0.227
	After 2nd listening		62.73 ± 21.97	60.73 ± 25.90	0.491	0.627
	Intra-group comparison	t	1.16	0.771		
		p	0.255	0.447		

^a^The scoring for the “Stress” dimension is reversed, with higher scores indicating lower levels of stress

^*^p < 0.05

## Discussion

The main objective of this study was to investigate the effects of broadband music and audible band music on relaxation states and cognitive function in young adults. It was found that the mean heart rate of the broadband group remained consistently lower than that of the audible band group throughout all eight time points, although these differences were not statistically significant. Furthermore, more significant differences were observed across time points within the broadband group than within the audible band group. Heart rate is regulated by the two branches of the autonomic nervous system (ANS): the sympathetic nervous system (SNS) and the parasympathetic nervous system (PNS), and it is an index of both SNS and PNS activity. The results of this study suggest that compared with listening to audible band music, listening to broadband music can lead to a higher relaxation level and a lower stress level, which may be attributed to increased PNS activity and decreased SNS activity [16]. This is consistent with previous research findings, which have demonstrated the potential benefits of music in promoting relaxation and reducing stress levels [17, 18]. The mechanism underlying this effect is thought to be mediated by the ANS, which regulates heart rate, blood pressure, and other bodily functions. Music can activate the PNS branch of the ANS, which promotes relaxation and slows down heart rate, while also inhibiting the SNS, which is responsible for the “fight or flight” response and can increase heart rate and blood pressure. In addition to its effects on the ANS, music has also been shown to activate the neural reward system [19] or the brain’s reward center [20], which can further promote relaxation and positive emotions, which is thought to be mediated by the release of dopamine and other neurotransmitters in the mesolimbic pathway that is associated with reward processing and pleasure.

Both the broadband and audible band group showed improvement in cognitive flexibility and executive function scores, which can be attributed to the positive effects of listening to music as reported by previous studies [21–25]. The mechanisms underlying the enhancement of cognitive function by music listening are complex and multifaceted, involving various neural and physiological processes. Studies have shown that listening to music can activate the dopaminergic system, which improves attention and motivation, as well as modulate neural plasticity, which enhances learning and memory [21]. Kirk et al. [22] found that listening to relaxing music reduced stress and improved cognitive performance in healthy aging adults, while Flaugnacco et al. [23] demonstrated that music-based interventions improved cognitive function in children with developmental disorders. However, the audible band group showed more significant improvements across time points compared to the broadband group in terms of cognitive flexibility and executive function, possibly because the participants in broadband group were more relaxed while listening to music, leading to drowsiness and reduced attentional focus and arousal levels, thereby affecting their performance in cognitive tests. According to Scheufele [24], relaxation can decrease attentional focus by reducing SNS activity and corresponding arousal levels, a process mediated by the interplay between SNS and PNS that regulate the body’s stress and relaxation responses. It is noteworthy that, in the absence of music intervention during Test 4 on Day 2, the participants exhibited continued improvement in cognitive function compared to the baseline. This phenomenon can potentially be attributed to the familiarity gained through the tasks administered on Day 1, enabling participants to handle the cognitive function tests more proficiently and accurately on Day 2.

In this study, the “D1B + D2A” group showed a significantly lower reaction time at pre-listening on Day 2 compared to after the second broadband listening session on Day 1 as well as after the first/second audible band listening session on Day 2. The reason might be listening to broadband music can indirectly shorten reaction time by improving sleep quality and emotional states. One mechanism for this may be through the regulation of the hypothalamic–pituitary–adrenal axis (HPA axis) [26], which plays a critical role in regulating sleep and emotions. Listening to broadband music can decrease cortisol levels, a hormone associated with stress and arousal, and increase levels of melatonin, a hormone associated with sleep regulation [27, 28]. This can result in better sleep quality, which has been shown to improve reaction time [29]. Another mechanism is through the reduction of anxiety and stress. Anxiety and stress have been shown to impair reaction time and other cognitive functions, such as attention and working memory [30]. Listening to broadband music has also been shown to improve mood and reduce anxiety, which can positively impact reaction time [31]. A study by Sakamoto et al. [32] found that interactive music interventions could decrease stress levels and restore residual cognitive and emotional functions in individuals with severe dementia, which holds promise for enhancing dementia patients’ quality of life. Moreover, the reduction of muscle tension and the corresponding improvement in motor coordination brought about by the higher level of relaxation after exposure to broadband music may also help to improve reaction time. Studies have shown that relaxation techniques, such as progressive muscle relaxation, can reduce muscle tension and improve motor coordination, leading to faster and more accurate movements [33]. Finally, relaxation can also improve reaction time by increasing the availability of cognitive resources. When individuals are relaxed, they tend to have improved attention and better cognitive resource allocation towards the task at hand, resulting in faster and more accurate responses [34].

The significant improvement in stress levels as self-reported by the participants using VAS during the second listening session compared to the first listening session in the broadband group can be explained by the reduced heart rates indicating increased PNS activity, higher relaxation levels, and lower stress levels, as discussed above.

These findings suggest that the significant within-group differences in executive function and cognitive flexibility only appeared at specific time points, likely due to the immediate but short-lived effects of music listening. Music can have immediate effects on cognitive functions and relaxation levels, which may explain the observed improvements right after the listening sessions that did not persist over a longer period, resulting in similar overall outcomes between the groups by the end of the study. Additionally, the participants’ familiarity with the tasks over time and the possibility of improved sleep quality might have contributed to these effects. As participants became more accustomed to the cognitive tasks, their performance could have improved due to practice effects, potentially overshadowing the specific effects of the two music types on cognitive flexibility and executive function. Furthermore, the short duration of our study might have limited our ability to observe long-term differences between the two groups. More extended exposure to the music types could potentially result in more pronounced and lasting differences in cognitive function and relaxation levels.

There are several limitations in this study. Firstly, the participants listened to both types of music only for a relatively short period of time (15 min/session, two sessions), which only allowed for an assessment of the immediate effects of music on cognitive function, rather than its long-term effects. Secondly, the music used in this study was soothing instrumental music played on the piano and harp. While it helped to relax the participants, it may have also caused drowsiness and reduced attentional focus, which could have affected their performance in cognitive tests. Thirdly, the study did not include a no-music control group, so our findings were limited to the comparison between the effects of short-term broadband and audible band music listening. In future studies, it may be worthwhile to consider using other types of music. Lastly, the study only included young adults aged 20–30 years old who did not have any cognitive impairments, which may have limited the potential for improvement in cognitive performance. Future studies could consider conducting long-term broadband music listening experiments with older adults who have cognitive impairments to determine the effects of broadband music on cognitive function in this population.

## Conclusion

Comparative analysis in our study showed that broadband and audible band music influenced cognitive function differently. Specifically, short-term listening to audible band music significantly improved cognitive flexibility and executive function, while short-term listening to broadband music significantly reduced reaction time in cognitive tests. The potential drowsy effect of the selected relaxing broadband music might have counteracted the possible improvement in cognitive flexibility and executive function. Therefore, it is necessary to explore the effects of broadband music using different music types to further validate its cognitive-enhancing effects. Additionally, broadband music showed a significant effect in reducing the heart rate of participants compared to audible band music, making them feel more relaxed and less stressed. Based on the current study results, future research can consider using broadband music to promote relaxation, shorten reaction time, and improve work efficiency. It can also be explored for its potential in preventing age-related cognitive decline and treating mild cognitive impairment in older adults.

## Data Availability

The data that support the findings of this study are available from the corresponding author upon reasonable request.

## Declarations

### Declarations

Ethical approval and consent to participate.

Research ethics approval was granted by the Institutional Review Board of Soochow University (SUDA20221228H07) in accordance with the principles of the Declaration of Helsinki. All participants signed an informed consent form before participating in the study.
